# Supplementary material for: Prognostic Role of TAPSE to PASP Ratio in Patients Undergoing MitraClip Procedure
Source: J Clin Med. 2021 Mar 2;10(5):1006. doi: 10.3390/jcm10051006 (PMC7958333; doi:10.3390/jcm10051006)
Supplement: Supplementary file 1 [file jcm-10-01006-s001.pdf]

Supplementary Table 1. Baseline characteristics according to the combined endpoint.

	No event (n=157)	Event (n=71)	p-value
**Baseline clinical characteristics**			
Age(years)	72.4±10.9	72.7±12.7	0.883
Sex, male(n,%)	106(67.5)	48(67.6)	0.989
Hypertension(n,%)	116(74.4)	49(69)	0.402
Diabetes mellitus(n,%)	42(26.8)	32(45.1)	0.006
Atrial fibrillation(n,%)	87(55.8)	46(65.7)	0.104
Glomerular filtration rate(ml/min/1.73m^2)	62.3±25.7	57.5±24.8	0.870
Stage 3b-5 chronic renal failure(n,%)	47(29.9)	28(39.4)	0.287
Ischemic heart disease(n,%)	79(50.3%)	34(47.9)	0.942
Prior PCI(n,%)	51(32.5)	25(35.2)	0.897
Prior CABG(n,%)	29(18.5)	13(18.3)	0.846
Extra cardiac arteriopathy(n,%)	22(14)	13(18.3)	0.572
Previous cardiac surgery(n,%)	45(28.7)	17(23.9)	0.461
COPD(n,%)	33(21)	16(22.5)	0.796
FC NYHA III-IV/IV(n,%)	131(83.4)	67(94.4)	0.024
EuroScore II	8.1±7.9	9.4±8	0.273
STS Score	5.3±4.7	7.1±6.4	0.059
NT-proBNP	5078.5±6721.9	4021.8±3721.4	0.226
**Baseline echocardiographic parameters**			
LVEDD(mm)	60.8±10.4	60.9±9.6	0.901
LVESD(mm)	49.3±13.4	50.9±10.9	0.461
Indexed LVEDV(ml/m^2)	91.3±31.5	94.3±41.4	0.588
Indexed LVESV(ml/m^2)	53.8±29.3	60.9±34.9	0.173
LVEF(%)	41.4±15.7	38.2±14.2	0.143
Indexed LA volume(ml/m^2)	59.2±23.6	62.8±25.6	0.384
MR grade IV/IV(n,%)	126(80.3)	61(85.9)	0.439

Regurgitant Volume(ml)	58.2±21.1	63.9±26.7	0.383
Effective Regurgitant Orifice(mm2)	43.6±18.2	41.7±15.9	0.609
Indexed LVEDV/ Effective Regurgitant Orifice(ml/mm2)	2.4±1.3	2.5±1.6	0.769
MR etiology(n,%)*			0.160
- Primary	39(25.2)	11(15.7)	
- Secondary	95(61.3)	52(74.3)	
- Mixed	21(13.5)	7(10)	
Tricuspid regurgitation(n,%)			0.424
- Grade 0-1	73(46.5)	25(35.2)	
- Grade 2	36(22.9)	21(29.6)	
- Grade 3	31(19.7)	15(21.1)	
- Grade 4	17(10.8)	10(14.1)	
TAPSE(mm)	17.4±4.2	16±4.4	0.017
PASP(mmHg)	48±14.7	50.9±13.8	0.176
TAPSE/PASP ratio	0.41±0.19	0.33±0.13	0.003
**Right heart catheterization** $^{\phi}$			
Right atrial mean pressure(mmHg)	10.3±6	6.9±3.7	0.116
LA mean pressure(mmHg)	17.9±8.4	26.9±29.2	0.213
Left atrial V-wave pressure(mmHg)	36.7±16.3	34.4±15.9	0.684
Change in LA mean pressure(mmHg)	4.9±7.2	8.1±6.5	0.203
Change in LA v-wave pressure(mmHg)	17.8±16.1	13.5±10.8	0.431
PCWP pressure(mmHg)	18.2±7.5	19.6±7.3	0.629
Mean PA pressure(mmHg)	28.7±10.2	32.9±13.9	0.389
Systolic PA pressure(mmHg) $^{\gamma}$	43.9±15.6	54±21.4	0.054
PA Pulse Pressure(mmHg)	23.6±6.8	30.9±15.9	0.218
Cardiac index(ml/min/m2)	2.1±0.6	2.3±0.7	0.303
Pulmonary vascular resistance(WU)	3.2±1.4	2.9±1.2	0.623
Transpulmonary pressure gradient(mmHg)	10.8±3.4	13.7±9.1	0.257

Diastolic pressure gradient(mmHg)	2.5±2.6	3.2±4.1	0.585
PA compliance(ml/mmHg)	2.4±0.9	2.6±1.7	0.680

*values for 225 patients. [ϕ]values for 26 patients. [ʏ]values for 51 patients.

CABG:coronary artery bypass graft. COPD:chronic obstructive pulmonary disease. LA:left atrial. LVEDD:left ventricular end-diastolic diameter. LVEDV:left ventricular end-diastolic volume. LVEF:left ventricular ejection fraction. LVESD:left ventricular end-systolic diameter. LVESV:left ventricular end-systolic volume. MR:mitral regurgitation. PA:pulmonary artery. PASP:pulmonary artery systolic pressure. PCI:percutaneous coronary intervention. PCW:pulmonary capillary wedge. STS:Society of Thoracic Surgery. TAPSE:tricuspid annular plane systolic excursion. WU:wood unit.

Supplementary Table 2. Procedural and 30-day outcomes according to the combined endpoint.

	No event (n=157)	Event (n=71)	Total sample (n=228)	p-value
Number of clips	1.5±0.6	1.4±0.7	1.5±0.6	0.517
Device time (minutes)	81.3±38.9	73.2±24.7	78.6±34.9	0.171
Procedural time (minutes)	144.3±52.2	142.4±69.6	143.7±57.7	0.846
Inotropic treatment (n, %)	16(10.2)	11(15.5)	27(11.8)	0.500
IABP (n, %)	8(5.1)	3(4.2)	11(4.8)	0.815
Postprocedural MR (n, %)				0.897
0-I/IV	100(63.7%)	47(66.2%)	147(64.5)	
II/IV	57(36.3%)	24(33.8%)	81(35.5)	
**Periprocedural complications and 30-day outcomes**				
Pericardial effusion (n, %)	1(0.6)	0	1(0.4)	0.500
Air embolism (n, %)	2(1.3)	0	2(0.9)	0.569
Cordal rupture (n, %)	1(0.6)	0	1(0.4)	0.689
Cordal entrapment (n, %)	1(0.6)	0	1(0.4)	0.689
Catheter thrombosis (n, %)	0	0	0	1.0
Clip detachment (n, %)	1(0.6)	0	1(0.4)	0.689
BARC bleeding (n, %)				0.148
- Type II	2(1.3)	2(2.8)	4(1.8)	
- Type III a-b	5(3.1)	1(1.4)	6(2.6)	
Access-site vascular complications (n, %)				
- Pseudoaneurism and AV fistula	3(1.9)	1(1.4)	4(1.8)	0.632
- Vascular surgery	4(2.5)	0	4(1.8)	0.222
Acute kidney injury (n, %)	1(0.6)	1(1.4)	2(0.9)	0.527
Stroke (n, %)	0	1(1.4)	1(0.4)	0.311
Pulmonary thromboembolism (n, %)	1(0.6)	0	1(0.4)	0.689
Gastrointestinal BARC 3a haemorrhage (n, %)	2(1.3)	0	2(0.9)	0.569

Nosocomial pneumonia (n, %)	0	2(2.8)	2(0.9)	0.096
In-hospital death (n, %)	0	2(2.8)	2(0.9)	0.035
30-day readmission for HF (n, %)	0	6(8.5)	6(2.6)	<0.001
Peri-procedural and 30-day complications (n, %)	17(10.8)	10(14.1)	27(11.8)	0.481

AV: arterio-venous. BARC: Bleeding Academic Research Consortium. HF: heart failure. IABP: intra-aortic balloon pump. MR: mitral regurgitation.

Supplementary Table 3. Follow-up TAPSE/PASP ratio determinations after successful transcatheter mitral valve repair.

	**Preprocedural TAPSE/PASP ≤0.35**	**Preprocedural TAPSE/PASP >0.35**	**p-value**
Postprocedural TAPSE/PASP ≤0.35	22(34.9)	10(16.4)	0.018
Postprocedural TAPSE/PASP >0.35	41(65.1)	51(83.6)	
Improvement in postprocedural TAPSE/PASP	55(87.3)	25(41)	0.001
Worsening in postprocedural TAPSE/PASP	8(12.7)	36(59)	

PASP: pulmonary artery systolic pressure. TAPSE: tricuspid annular plane excursion.
